# Recent Advances in the Discovery of Novel Antiprotozoal Agents

**DOI:** 10.3390/molecules24213886

**Published:** 2019-10-28

**Authors:** Seong-Min Lee, Min-Sun Kim, Faisal Hayat, Dongyun Shin

**Affiliations:** College of Pharmacy, Gachon University, 191 Hambakmoe-ro, Yeonsu-gu, Incheon 21936, Korea; minlee0405@gmail.com (S.-M.L.); ms9404@hanmail.net (M.-S.K.); faisalchem80@yahoo.com (F.H.)

**Keywords:** protozoan diseases, parasitic, giardiasis, leishmaniasis, malaria, trichomoniasis, trypanosomiasis

## Abstract

Parasitic diseases have serious health, social, and economic impacts, especially in the tropical regions of the world. Diseases caused by protozoan parasites are responsible for considerable mortality and morbidity, affecting more than 500 million people worldwide. Globally, the burden of protozoan diseases is increasing and is been exacerbated because of a lack of effective medication due to the drug resistance and toxicity of current antiprotozoal agents. These limitations have prompted many researchers to search for new drugs against protozoan parasites. In this review, we have compiled the latest information (2012–2017) on the structures and pharmacological activities of newly developed organic compounds against five major protozoan diseases, giardiasis, leishmaniasis, malaria, trichomoniasis, and trypanosomiasis, with the aim of showing recent advances in the discovery of new antiprotozoal drugs.

## 1. Introduction

It is well known that parasitic diseases are a serious health problem, that has a deep impact on the global human population [1]. Among parasites, protozoan parasites, such as *Trypanosoma cruzi*, *Leishmania mexicana*, *Plasmodium falciparum*, *Giardia intestinalis*, and *Trichomonas vaginalis*, are the major disease-causing organisms. They are responsible for spreading infections worldwide, especially in undeveloped countries, where a tropical or temperate climate and poor sanitary and hygiene conditions are common [2,3,4]. Protozoan parasites are single-celled eukaryotes characterized as a diverse polyphyletic group. The infections caused by these parasites are responsible for 500 million deaths worldwide [5,6,7,8]. These infections are considered neglected because relatively little attention has been devoted to their surveillance, prevention, and treatment [9]. According to the global burden of disease analysis report by the World Health Organization in 2008, around 17% of deaths worldwide are caused by neglected tropical diseases [10]. These neglected protozoan diseases are a group of tropical infections that are particularly dominant in low-income majority populations, and affect millions of people and animals [6,11]. The major neglected protozoan diseases are Chagas disease, leishmaniasis, trichomoniasis, amebiasis, and giardiasis [9,12,13].

The modes of transmission of these protozoan parasites differ from each other. Some of them are transmitted by insects that are vectors of *Plasmodium* species (malaria), *T. brucei* (human African trypanosomiasis, HAT), *T. cruzi* (Chagas disease), and *Leishmania* species (leishmaniasis). In addition, *E. histolytica* (amebiasis), *Cryptosporidium parvum* (cryptosporidiosis), *Cyclospora cayetanensis* (cyclosporiasis), and *Giardia lamblia* (giardiasis) are transmitted through food and water contaminated with fecal matter [12]. The biochemistry of a large number of protozoan parasites has been studied during previous studies. According to the mode of infection of the parasites, many organic molecules have been manufactured in the last 50 years for the development of new antiprotozoal agents. Some of these agents, e.g., metronidazole and tinidazole (for amebiasis, trichomoniasis, and giardiasis), sodium stibogluconate (for leishmaniasis), chloroquine (for malaria and trichomoniasis), pentamidine and melarsoprol (for HAT), and benznidazole (for American trypanosomiasis) are very effective and are currently used in medical practice. However, the available drugs are not the final solution because of their existing resistance and toxicity [14,15,16,17,18,19,20,21,22,23,24,25,26,27,28,29,30]. The above-mentioned shortcomings promote the ongoing drug research effort to identify mechanistically novel, nontoxic, and cost-effective chemotherapies for the treatment of these neglected protozoan problems [31]. 

As a continuation of our studies on antiprotozoal drug development, we present the latest information with respect to recent drug developments against five major protozoan diseases, giardiasis, leishmaniasis, malaria, trichomoniasis, and trypanosomiasis in the form of a review. Notably, during the literature review, we found many articles on antiprotozoal drug discovery published between 2012 and 2017 [32,33,34,35,36,37,38,39,40,41,42,43,44,45,46,47]. Therefore, keeping their findings in mind, we wrote this review in a different style; we have described the five major protozoan diseases and compiled their latest synthetic and pharmacological data individually from 2012 to 2017 in Table 1, Table 2, Table 3, Table 4 and Table 5. This tabular form of data has not been reported in previously published reviews.

## 2. Recent Progress of Antiprotozoan Agents

### 2.1. Anti-Giardiasis

Giardiasis is caused by the protozoan parasite *G. intestinalis* (also known as *G. lamblia* or *G. duodenalis*). It is also known by the common name “beaver fever,” because this infection was reported in campers who drank contaminated water that was inhabited by beavers. Giardiasis is the most common protozoan infection in human beings, and it occurs in both developing and industrialized countries [48]. Its global incidence is believed to range between 20%–60% [49], with 2%–7% incidence in industrialized nations [50]. *Giardia intestinalis* was first described in 1681 after the Dutch microscopist Antonie van Leeuwenhoek observed the protozoan in his own diarrheic stools. This disease is often prevalent in poor countries and communities that have untreated water, inadequate sanitation, and poor dietary status [51]. The infection is caused by fecal–oral transmission and initiated by the ingestion of infectious cysts from contaminated water or through person-to-person contact. After excystation, flagellated trophozoites colonize the upper small intestine, where they attach to the epithelial lining but do not invade the mucosa. The duration of Giardia infection is variable; however, chronic infection and reinfection commonly occur [52]. Approximately 50% of the symptoms classically associated with giardiasis are asymptomatic including diarrhea, abdominal pain, nausea, vomiting, and anorexia. However, infected individuals can also develop extraintestinal and postinfectious complications [53,54]. Chronic extraintestinal sequelae may affect the joints, skin, eyes, and central nervous system, but the underlying mechanisms are unknown [53,54]. According to research data, Giardia has eight distinct genetic assemblages labelled as assemblage “A” through “H” [55,56], and assemblages “A” and “B” are responsible for infection in humans.

**Figure 1:**
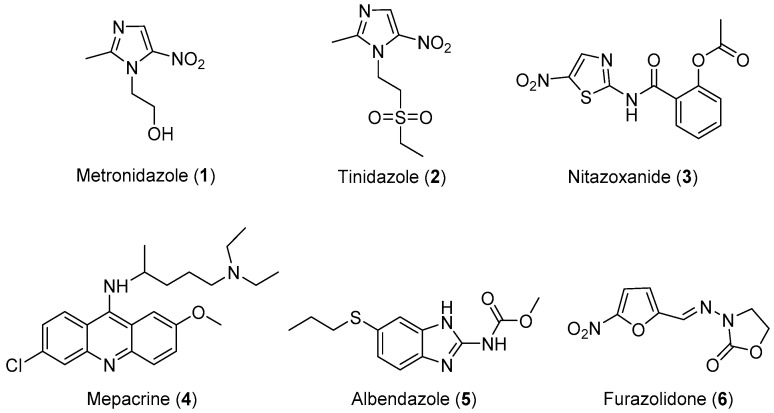
Currently available antigiardial drugs.

Three classes of drugs are currently used for the treatment of giardiasis: metronidazole, mepacrine analogs, and nitrofurans, such as furazolidone (Figure 1). Metronidazole is the most widely used drug for the treatment of giardiasis globally and it is generally effective and well-tolerated. However, the United States Food and Drug Administration (FDA) has not yet approved this drug for the treatment of Giardia infection because of its toxicity and major side effects, such as seizures, ataxia, peripheral neuropathy, transient myopia, gastric mucosal irritation, sperm damage, and hematuria [14,15,16,17,18]. Tinidazole is an N1-position modified 5-nitro imidazole, which has been approved by the FDA for the treatment of giardiasis. Nitazoxanide (NTZ) belongs to an emerging class of 5-nitrothiazole compounds with potential antigiardial activity [57]. However, although NTZ is generally well tolerated, some adverse effects such as abdominal pain, diarrhea, and nausea limit the safe use for human beings. Mepacrine is no longer available in the United States, and it is being replaced in most of its applications with safer and more specific drugs. Furazolidone also has serious side effects, such as gastrointestinal disturbances, hemolytic anemia, disulfiram-like reactions to alcohol, and hypersensitivity reactions, as well as evidence of tumorigenicity in rodent studies. Furthermore, *G. lamblia* resistance to this drug has also been reported [58,59,60]. Therefore, research focusing on the development of novel, alternative drugs for the treatment of giardiasis is highly desirable. In view of these considerations, researchers have designed and synthesized some novel molecules for the treatment of giardiasis, their results are summarized in Table 1.

#### Important Highlights of Table 1 Compounds

All of the novel compounds designed for giardiasis treatment are listed in Table 1 and their efficacies were compared with those of the standard drugs metronidazole (MTZ), formononetin, aminitrozole, nitazoxanide, tizoxanide, nitazoxanide and mebendazole. In brief, Zhang et al. isolated 3,5-dicaffeoylquinic acid from *Artemisia argyi*, and from it, developed a series of ester derivatives as potential antigiardial agents. Amongst the synthesized compounds, Compound **7** was reported as the most potent inhibitor against *G. lamblia* (IC_50_ = 4.62 µg/mL) [61]. A series of 3-tetrazolylmethyl-4H-chromen-4-ones were synthesized by Cano et al. via an Ugi-azide multicomponent reaction and evaluated for antigiardial activity and, compound **8** was found to be the most potential antigiardial agent (IC_50_ = 84.2 µg/mL) in the series [62]. Navarrete-Vázquez et al. [63] synthesized a series of four 5-nitrothiazole compounds and reported them as novel antigiardial agents. Among them, compounds **9a** (IC_50_ = 0.122 µM) and **9b** (IC_50_ = 0.151 µM) exhibited potential inhibitory activity against *G. intestinalis.* Singh et al. [64] designed and developed a series of chalconyl blended triazole allied silatranes; these compounds are hybrids of three pharmacologic scaffolds, namely chalcone, triazole and metal complex (silatranes). All the derivatives were evaluated for the antigiardial activity; among them compound **10** showed excellent activity against *G. lamblia* (IC_50_ = 19.58 µM). Compound **11** is a derivative of naturally occurring sesquiterpene lactone, which was isolated from *Decachaeta incompta* by Bautista et al. It showed greater antigiardial activity (IC_50_ = 30.6 µg/mL) than its parent compound [65]. Novel nitazoxanide–*N*-methylbenzimidazole hybrids were designed and synthesized by Soria-Arteche et al. [66], and evaluated for their in vitro biological activity. Compounds **12a–d** expressed good antigiardial activity (IC_50_ = 0.021–0.027 µM) by inhibiting an *G. intestinalis* culture. Using a novel methodology based on a double Sonogashira coupling reaction in 2-amino-3,5-diiodopyridine, Leboho et al. synthesized a series of 2,3,5-trisubstituted 7-azaindoles, as well as 2,5-disubstituted 7- azaindoles. These synthesized series were evaluated against a *G. duodenalis* strain, and the results showed that compounds **13a** (IC_50_ = 14.3 µg/mL) and **13b** (IC_50_ = 8.2 µg/mL) were the most potent agents [67]. Another series of 2-amino-4-arylthiazole derivatives were prepared and evaluated by Mocelo-Castell et al. [68] as potential anti-giardial agents. The results revealed that compounds **14a** (IC_50_ = 0.87 µM) and **14b** (IC_50_ = 0.39 µM) were the most potent inhibitors of *G. intestinalis*. Disulfiram (compound **15**) was proposed to inactivate *G. lamblia* kinase, and Castillo-Villanueva et al. hypothesized that it acts on enzymes of *G. lamblia*. Accordingly, compound **15** (IC_50_ = 6.6 µM) was efficient inactivator of immunoreceptor tyrosine-based inhibition motif (ITIM). Therefore, it is feasible that compound **15** could lead to new pharmacotherapies against *G. lamblia* [69]

In summary, the reported antigiardial agents could be categorized as the natural compounds and their analogues (e.g., 3,5-dicaffeoylquinic acid derivatives and 8-acyl and 8-alkyl incomptine A derivatives) and hybrids compounds with the known acitve drug such as nitazoxanide-based and benzimidazole-based hybrids. It is noteworthy that the hybrid of nitazoxanide and *N*-alkylbenzimidazole tethered by amide linker exhibited good activity profiles compared with nitazoxanide or albendazole, which suggests that hybridization of active compounds could provide good option for antigiardial drug discovery. 

### 2.2. Anti-Leishmaniasis

Leishmaniasis, a parasitic disease spread by the bite of an infected female phlebotomine sand fly, has been known for many years it was first clinically described in 1756 by Alexander Russell, who named it Aleppo boil [70]. Many names correlate to this group of diseases such as kala-azar, Dum-dum fever, white leprosy, espundia, and pian bois. A vector borne disease, leishmaniasis is caused by an obligate intramacrophage protozoan, and it is characterized by its diversity and complexity [71,72]. Approximately 21 *Leishmania* species have been identified to be pathogenic to humans. *Leishmania* is one of several genera within the family Trypanosomatidae, and its species are characterized by the possession of a kinetoplast, a unique form of mitochondrial DNA. Based on species type host immune system responses, leishmaniasis has three basic clinical forms: Cutaneous (with skin ulcers), mucocutaneous (with skin, mouth, and nose ulcers), and visceral (with liver, and spleen enlargement, as well as bone marrow dysfunctions) [73,74]. Cutaneous leishmaniasis, the most common form of the disease, is caused by *L. braziliensis*, *L. major*, *L. mexicana*, *L. tropica*, and several other species [75]. It can be eventually defeated by the immune system; however, in most cases it progresses and is converted in to the mucocutaneous form, in which the parasites metastasize to the mucosal tissues. Mucosal leishmaniasis is usually caused by *L. braziliensis*, and it is associated with damage to the palate, nasal septum, and mucous membranes [76,77]. This form is usually refractory to therapy and can be fatal. The most dangerous form of the disease is visceral leishmaniasis, commonly called kala-azar. It can become fatal if it is not rapidly diagnosed and treated, it is responsible for most leishmaniasis-related deaths [78,79]. In visceral leishmaniasis, which is caused by *L. donovani* and *L. infantum* (synonym of *L. chagasi*), the parasite mainly infects the liver, spleen, and bone marrow. The infected host shows symptoms such as fever, weight loss, and anemia [80]. This disease has been recognized as an increasing health problem worldwide by the World Health Organization (WHO) [81], with high morbidity and mortality rates in Africa, Asia, and America. Among all tropical diseases, leishmaniasis is ranked fourth in morbidity and second in mortality rates [82]. Leishmaniasis is widespread, having been reported in 88 countries across all continents, with the exception of Antarctica [71,83]. It has an annual death rate of approximately 80,000 people [84], and there are two million new cases occurring annually, with 12 million people currently infected globally [83,85].

**Figure 2:**
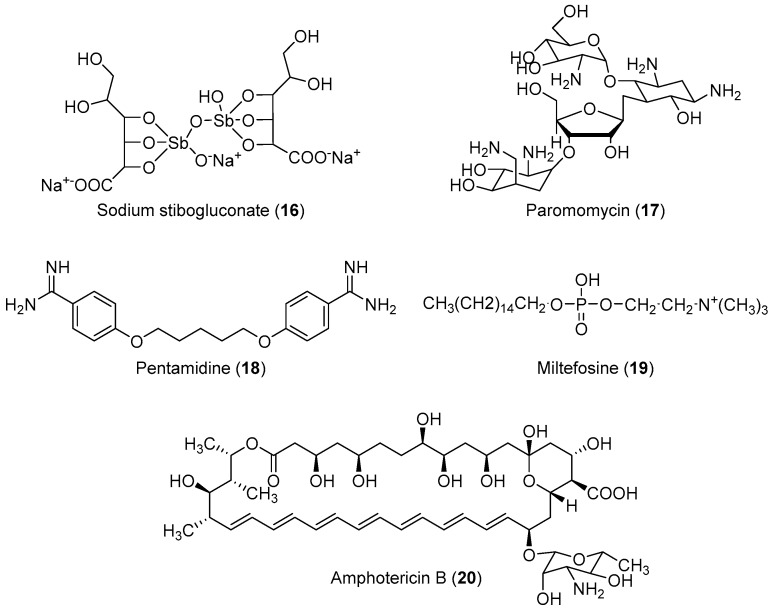
Currently available antileishmanial drugs.

The life cycle of *Leishmania sp.* begins when the invertebrate host (sand fly) feeds on infected mammalian blood, thereby imbibing the amastigotes present within the macrophages. In the intestine of the insect vector, the amastigote transforms into procyclic promastigotes, and later into metacyclic promastigotes. When the insect bites a mammalian host again, the inoculated virulent promastigotes enter the blood stream and are internalized by the macrophages, where they differentiate again into amastigotes, completing the cycle [86]. No effective vaccine is available against leishmaniasis; chemotherapy is the only effective way to treat all forms of the disease [87,88,89]. However, some drugs are available for the treatment of this disease. The first-choice treatment for leishmaniasis involves the use of the pentavalent antimonial derivatives, sodium stibogluconate, which is highly toxic with serious side effects, and requires a prolonged treatment regimen [90,91]. Alternatives include paromomycin, pentamidine, miltefosine and amphotericin-B (Figure 2) However, these drugs have not found extensive use owing to their severe toxicities and difficulties associated with parenteral administration and drug resistance [19,20,21,22]. Generally, the drugs currently used for the treatment of human cutaneous and visceral leishmaniasis are toxic, and can cause severe adverse reactions such as pancreatitis, pancytopenia, reversible peripheral neuropathy, nephrotoxicity, cardiotoxicity, bone pain, and myalgia [92,93]. Therefore, the development of novel, effective, and safe antileishmanial agents with reduced side effects is a major priority for health researchers. In view of these considerations, some researchers have designed and synthesized novel molecules for the treatment of leishmaniasis; their results are summarized in Table 2.

#### Important Highlights of Table 2 Compounds

Compounds of different heterocycles classes are included in Table 2 as potent antileishmanial agents and the results of their analysis were compared with those of the standard drugs pentamidine, sodium stibogluconate (SSG), miltefosine, amphotericin-B (AmB), edelfosine and clofazimine (CFM).

In brief, a series of thiosemicarbazide derivatives were prepared and evaluated for thier antileishmanial activities. Compounds **21a** and **21b** were reported as the most active candidates, with IC_50_ values in the range of 16.4 µM to 22.0 µM, after screening the whole series against *L. amazonensis* cultures using promastigotes and amastigote assays [94]. Among the compounds developed by Rashid et al., compound **22** was found to be a highly active antileishmanial agent. Basically, compound **22** is a designed hybrid compound in which two biological scaffolds, pyrazoline and pyrimidine are linked with each other. It showed excellent biological activity the highest among all theh hybrids in its series against *L. major* and *L. donovani* with IC_50_ values of 0.47 ± 0.02 and 1.5 ± 0.17 µg/mL, respectively [95]. Sangshetti et al. [96] synthesized 4,5,6,7-tetrahydrothieno[3,2-c]pyridine-based hydrazone derivatives and determined their antileishmanial inhibitory activities. Among the derivatives in this series, compounds **23a** and **23b** showed significant biological activities against *L. donovani* promastigotes, IC_50_ values of 98.75 and 93.75 µg/mL, respectively compared to that of the strandard drug SSG (IC_50_ = 490 µg/mL). A series of chalcones are also included in Table 2, and their activity against *L. donovani* cultures clearly showed that among them, the compounds containing chromane, pyridine, and a substituted 4-hydroxy phenyl ring (compounds **24a** and **24b**) exhibited excellent antileishmanial activities (IC_50_ values of 2.8 and 2.0 µM, respectively) [97]. Among a series of carboline derivatives reported by Manda et al. [98], compounds **25a** (IC_50_ = 12.7 µM) and **25b** (IC_50_ = 9.1 µM), which are derivatives of the commercially available tetrahydro-β-carboline prepared in a single procedure were the best candidates against *L. donovani* (promastigotes). Zhu et al. [99] reported compounds **26a** and **26b**, which are derived from arylamidamide using the reaction between amino diarylfurans and 2-pyridyl thioimidate analogs, as the most active antileishmanial agents against both intracellular *L. donovani* and *L. amazonensis* amastigotes, with IC_50_ values ranging from 0.13 to 0.31 µM. A series of 4-alkapolyenylpyrrolo[1,2-a]quinoxaline derivatives, including compounds **27a** and **27b**, which exhibited remarkable inhibitory potential against two *Leishmania* spp. strains namely *L. major* and *L. donovani* (IC_50_ values between 1.2 and 10.5 µM), were prepared and reported by Ronga et al. [100]. Of all the compounds synthesized by Bekhit et al. [101], compound **28**, whose hybrid analog baasically resulted from hybridization with five-membered heterocyclici moieties, including 1,3,4-thiadiazoles and pyrazolines, exhibited the greatest potential in terms of antileishmanial activity. It was the best candidate among the series against *L. aethiopica* promastigotes (IC_50_ = 0.0142 µM) and amastigotes (IC_50_ = 0.13 µM). Compound **29**, an acetamide derivative of (+) -dehydroabietylamine derivative reported by Dae-ayuela et al [102], was found to be the most potent leishmanicidal agent among the series (IC_50_ = 1.5 µM). It was even more active than the reference compound, miltefosine (IC_50_ = 3.4 µM). A series of 2-phenyl-3-(pyridin-4-yl)imidazo[1,2-a]pyrazine derivatives were synthesized by Marchand et al. [103], and their antileishmanial activities were evaluated. Among the synthesized molecules, compounds **30a** and **30b** were found to be the most potent analogs against *L major* promastigotea and amastigotes with IC_50_ values between 0.2 and 6.4 µM. Sangshetti et al. [105] synthesized a series of indolyl–coumarin hybrids, and after evaluating their antileishmanial potential in vitro reported that compounds **31a** and **31b** were excellent antileishmanial agents (IC_50_ values between 95 and 99 µg/mL) compared to the standard drug SSG (IC_50_ = 490 µg/mL) [104]. The sntileishmanial potentials of 1,3,6-trisubstituted β-carboline derivatives, synthesized by Lunagariya et al. were evaluated, and compound **32** (IC_50_ = 9.0 µM) was found to show an comparable antileishmanial activity comparable to that of the standard drug, miltefosine (IC_50_ = 11.9 µM). Kumar et al. [106] reported the synthesis of a new series of aryl substituted ketene dithioacetals, which were evaluated in vitro for their activity against *L. donovani*. Based on their results, compounds **33a** and **33b** were reported as the most potent antileishmanial agents, with IC_50_ values of 5.12 and 3.56 µM respectively. Among the 4-arylamino-6-nitroquinazolines synthesized by Saad et al. [107], compounds **34a** and **34b** were found to be the most potent inhibitors of *L. major* promastigotes (IC_50_ values of 1.87 and 4.37 µM, respectively) compared to the standard drug, pentamidine (IC_50_ = 5.09 µM). Gopinath et al. [108] developed a series of substituted quinoline analogs and assessed their antileishmanial activities. They found compound **35** to be the most active (IC_50_ = 0.84 µM). Among the diselenide and sulfonamide derivatives developed by Baquedano et al. [109] compounds **36a–c** were found to be potent antileishmanial agents, with IC_50_ values of 1.40, 1.47 and 0.83 µM, respectively, against *L. infantum* intracellular amastigotes. An assessment of the antileishmanial potential of triazolopyridyl pyridyl ketone derivatives developed by Adam et al. [110] revealed that compounds **37a** and **37b** elicited potent growth inhibition against cultured *Leishmania* spp. promastigotes and amastiogotes with IC_50_ values ranging between 19.5 and 114.6 µM. Sharma et al. [111] synthesized Triazino indole–quinoline hybrid as antileishmanial agents targeting *L. donovani*. Their results showed that compounds **38a** and **38b** significantly inhibited *L. donovani* extracellular promastigotes and intracellular amastigotes with IC_50_ values ranging between 0.36 and 8.57 µM. Among the heteroretinoid-bisbenzylidine ketone hybrids developed by Tiwari et al. [112], compounds **39a–c** were identified as the most potent agents against *L. donovani* intramacrophagic amastigotes, with IC_50_ values between 1.83 and 5.02 µM. Pandey et al. [113] synthesized indole-2-carboxamide derivatives using utilizing the isocyanide based multicomponent reaction and evaluated them against *L. donovani*. Their results showed that among them, compound **40** (IC_50_ = 0.6 µM) exhibited a more promising antileishmanial activity than those of standard drugs, including SSG (IC_50_ = 56.1 µM) and miltefosine (IC_50_ = 8.4 µM). Several Clofazimine analogs were synthesized by Barteselli et al. [114] and their antileishmanial activities were screened using an in vitro evaluation, which demonstrated that compound **41** was the most potent antileishmanial agent against *L. infantum*, and *L. tropica*. A series of 8 imidazole derivatives were developed by Vita et al. [115], and an in vitro analysis of their antileishmanial activities showed that out of the 8 compounds, compounds **42a** and **42b** were the most potent antileishmanial molecules with IC_50_ values of 12.7 and 8.0 µM against *L. infantum* respectively. Reddy et al. [116] synthesized a large series of benzyl phenyl ether derivatives and evaluated their biological activities. Their results showed that compounds **43a–e** were potent antileishmanial agents and compounds **43b** (IC_50_ = 1.6 µM), **43d** (IC_50_ = 1.27 µM) and **43e** (IC_50_ = 1.39 µM) showed the greater antileishmanial activity against *L. donovani* compared to that of the standard drug, pentamidine (IC_50_ = 1.84 µM). Zhang et al. [117] prepared a novel series of oxyneolignans virolin, surinamensin, and analogs using an asymmetric synthetic method. Thereafter, their ability to inhibit the growth of different protozoal strains was tested. Their results showed that compounds **44a** and **44b** exerted the maximum antileishmanial activities with IC_50_ of 2.29 µg/mL and 2.48 µg/mL, respectively. The antiprotozoal activities of novel oxadiazolyl pyrrolo triazole diones derivatives synthesized by Dürüst et al. [118] was investigated. The results showed that compounds **45a** (IC_50_ = 1.6 µg/mL) and **45b** (IC_50_ = 2.0 µg/mL) were the most active antileishmanial agents against *L. donovani*. Pierson et al. [119] synthesized a series of novel 4-arylcoumarin derivatives, and the determination of their antiprotozoal activities against various biological strains revealed that compounds **46a–c**, with different substitutions on phenyl rings, displayed the most potent activity against *L. donovani* amastigotes (IC_50_ = 1.1–5.4 µM). Patric et al. [120] developed a series of bis-pyridylimidamide derivatives, and an assessment of their antileishmanial activities showed that among them, compound **47a** was the most active, and inhibited the *L. amazonensis* strain with an IC_50_ value of 0.095 µM, while the others, including **47b** (IC_50_ = 0.123 µM) and **47c** (IC_50_ = 0.211 µM), exhibited slightly more potent activity compared with amphotericin B (IC_50_ = 0.124 µM). Diaryl sulfide inhibits *L. infantum* promastigotes, and an evaluation of the inhibitory activity of its deriatives by Saccoliti et al. [121] showed that compound **48** inhibited *L. infantum* promastigotes by a dose-dependent amount with an IC_50_ value of 29.43 µM. Preeti et al. [122] developed tellurium derivate, immunomodulatory, and demonstrated that compound **49** could eliminate *L. donovani* promastigotes, and an evaluation by in vitro assay showed that it had a significant growth inhibitory effect on *L. donovani* promastigotes with an IC_50_ value of 26.9 µM. Rodríguez-Hernandez et al. [123] converted hederagenin into 1,2,3-trizolyl derivatives aiming to obtain antileishmanial and cytotoxic compounds. Of the synthesized compounds, compound **50** was found to be the most potent antileishmanial molecule, with an IC_50_ value of 5.6 µM. The thiadazole scaffold is a prevalent heterocyclic ring with antiparasitic activity. Tahghighi et al. [124] developed its derivatives, among which compounds **51a** and **51b** (IC_50_ between 0.08=9.35 µM) were found to be the most potent antileishmanial agents inhibiting extracellular promastigotes and amastigotes. 

In summary, antileishmanial agents of diverse scaffolds such as chalcone, arylamidine, thiohydrazone, and polyheteroaromatics, were reported. Som of the compounds exhibited comparable activity with pentamidine or miltefosine. In particular, 1,5-diphenylpenta-1,4-dien-3-one derivatives (**39**) and Ether-tether phenylamidine (**43**) displayed good activity profiles.

### 2.3. Anti-Malaria

**Figure 3:**
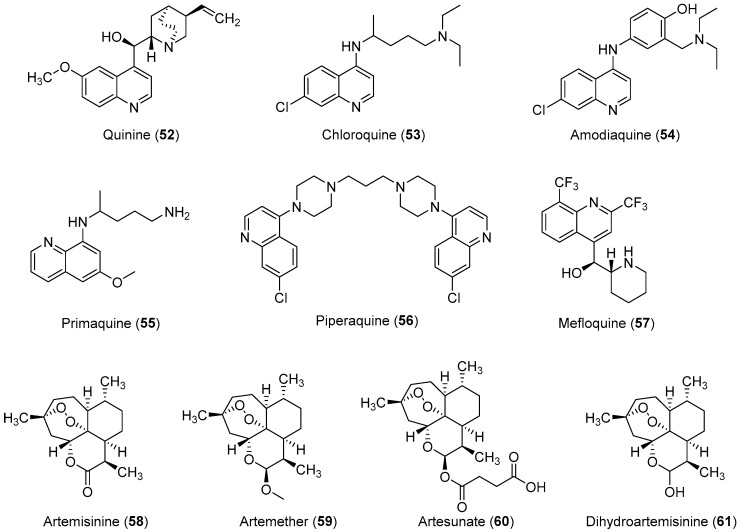
Currently available antimalarial drugs.

Malaria is a deadly mosquito-borne disease that mainly affects humans. It is an infectious disease caused by protozoan parasites belonging to the genus *Plasmodium*. Five different species of *Plasmodium*, *P. falciparum*, *P. ovale*, *P. malariae*, *P. vivax*, and *P. knowlesi* are responsible for the spread of malaria; *P. falciparum* is considered the most dangerous and virulent form. The disease is transmitted via the sucking of human blood by infected female *Anopheles* mosquitoes [125], and symptoms include fever, fatigue, vomiting, and headache; in severe cases, it can cause yellow skin, seizures, coma, and death [126]. The symptoms usually begin 10 to 15 days after infection, and disease recurrence may be observed months later if not properly treated. In those who have recently survived an infection, reinfection usually causes milder symptoms. This partial resistance disappears over months to years if the person has no continuing exposure to malaria [126]. According to the 2014 world malaria report by the WHO, a total of 198 million cases of malaria and nearly 584,000 malaria deaths occurred in 2013 [127]. In 2013, 3.4 billion people were at risk of malaria, of whom 1.2 billion were at a higher risk with more than one case per 1000 people, especially in over 97 countries in tropical areas with the ongoing transmission of malaria. Ninety percent of the above-mentioned deaths occurred in sub-Saharan Africa, of which 77% were children under the age of five. In 2010, an estimated 660,000 malaria deaths were reported worldwide [128]. Additional reports suggest that malarial infection and mortality are more widespread than previously estimated by the report of Murray et al., with up to 200 million clinical cases and 1.2 million deaths reported in 2010 alone [129]. Because of its devastating effects on the human population, the WHO rates malaria as one of the top three infectious diseases worldwide [130]. In brief, malaria has become one of the major causes of illness in humans, with approximately 250 million clinical cases reported around the world annually, particularly in poor or developing countries [127]. *P. falciparum* causes most of the severe cases and the most deaths, and nearly 80% of all reported cases and as mentioned above 90% of malaria-attributed deaths occur in Africa [131]. Moreover, *P. vivax* is the predominant species, in Asia, the Middle East, Central and South America, and the Western Pacific [132]. Malarial parasites exhibit a complex life cycle involving an insect vector (mosquito) and a vertebrate host (human), and all species exhibit a similar life cycle with only minor variations. The infection is initiated when sporozoites are injected into the human body through the saliva of a feeding *Anopheles* mosquito. When sporozoites enter the human body and travel through the bloodstream to the liver they transform into exoerythrocytic forms (EEFs) [133]. Based on the *Plasmodium* species, these forms are converted into mature exoerythrocytic-stage schizonts, or enter a dormant phase in which they are called hypnozoites, which only two species of *Plasmodiums*, *P. vivax* and *P. ovale* make. These hypnozoites reactivate several weeks to months (or years) after the primary infection and are responsible for malaria relapses weeks, months, or even years after the initial infection [134]. Fully developed exoerythrocytic-stage merozoites eventually exit the liver and re-enter the bloodstream [133]. They enter the red blood cells (RBCs) and replicate asexually causing RBC destruction, which leads to the characteristic symptoms associated with malaria such as anemia, fever, and chills [135]. A small percentage of these asexual blood-stage parasites then differentiate into sexual erythrocytic stages (female and male gametocytes) whose transmission back to the mosquito vector during a subsequent blood meal completes the life cycle [136]. Important antimalarial agents are presented in Figure 3. In the past, malaria was treated with the bark of cinchona (*Cinchona rubra* [*Rubiaceae*]); however, at this time, it was not known that cinchona bark contains quinine, which was later isolated and shown to have antimalarial properties [137,138]. Some medicinal chemists developed simpler synthetic analogs of quinine such as chloroquine, amodiaquine, primaquine, and piperaquine, which all had a quinine pharmacophore, but did not have multiple stereogenic centers. Among them, chloroquine was found to be the most efficient drug, and it has served humanity for over five decades [42,139]. However, the spread of chloroquine resistance prompted medicinal chemists to re-investigate the chemistry and pharmacology of alternative 4-aminoquinoline antimalarials such as amodiaquine [132], which has proven to be effective against chloroquine-resistant parasite strains [23,24,25,26,27]. Amodiaquine is effective against many chloroquine-resistant strains of *P. falciparum* [126]. However, its clinical use has been severely restricted because of its associations with hepatotoxicity and agranulocytosis [28,29]. Although in recent years a natural endoperoxide artemisinin and its semisynthetic derivatives artemether, artesunate, and dihydroartemisinin have been employed for the treatment of malaria owing to chloroquine resistance in parasites, but the global deployment of artemisinin-based combination therapy is limited by its relatively high cost of treatment, safety concerins during pregnancy, and early signs of resistance in Southeast Asia [140,141,142]. Despite the market availability of large numbers of antimalarial drugs, no perfect drug is known because individual drugs and drug combinations have their own limitations including poor compliance, side effects, toxicity, and resistance. Therefore, owing to the aforementioned conditions, some researchers have designed and synthesized novel molecules for antimalarial purposes. In this review article, we have compiled the latest data on antimalarial agents designed from 2012–2017. The details of these antimalarial agents are summarized in Table 3.

#### Important Highlights of Table 3 Compounds

Compounds of different heterocycle classes are included in Table 3 as potent antimalarial agents and the results of their assessment were compared to those of the standard drugs dihydroartemisinine, chloroquine, artemisinin, mefloquine, puromycin, artesunate, artemether, artesunic acid, artemisinin derived alcohol, di-artemisinin, proguanil, and quinone.

Pingaew et al. [143] synthesized 11 chalcone coumarin hybrids linked by the 1,2,3-triazole ring. All the derivatives were screened, and the evaluation of their potent antimalarial activity showed that among them, compound **62** significantly inhibited a *P. falciparum* culture with an IC _50_ value of 1.60 µM. Nisha et al. [144] developed β-amino-alcohol tethered 4-aminoquinoline-isatin conjugates, and evaluation of their antimalarial activities revealed that compounds **63a** and **63b** were the most potent antimalarial agents, with IC_50_ values of 11.7 and 13.5 nM respectively. Kumar et al. [145] synthesized various triazole tethered isatin-ferrocene derivatives and evaluated their antimalarial activities against chloroquine-susceptible and chloroquine-resistant *P. falciparum* strains. The results showed that compounds **64a** and **64b** were potent antimalarial agents. Several aminoalkylated quercetin derivatives were synthesized by Helgren et al. [146] by using the mannich reaction were screened for antimalarial activity using an in vitro assay. The results demonstrated THAT compounds **65a** and **65b** were the most potent antimalarial agents against three drug-resistant malarial strains (D6 and W2), with IC_50_ values between 0.065 and 0.079 µM. A series of fosmidomycin analogs were developed by Phillips et al. [147] and analyzed in vitro. The results showed that of all the synthesized compounds, compound **66** was the most potent antimalarial molecule with an IC_50_ values of 27.4 nM against chloroquine- and mefloquine-resistant Dd2 strains of *P. falciparum*. Yadav et al. [148] synthesized a series of marine-derived indole alkaloid derivatives, and an evaluation of their biological activities showed that compound **67** was the most potent antimalarial agent. Le et al. [149] designed a novel series of 11-aza-artemisinin analogus and tested them to determine their ability to inhibit the growth of FcB1 strains of *P. falciparum*. The results showed that compounds **68a–c** exerted the greatest antimalarial activities with IC_50_ values of 0.3, 0.7, and 1.5 µM respectively. Two dimers and two trimers of artemisinin hybrids were synthesized by Reiter et al. [150] and their antimalarial activities were investigated using an antimalarial assay against *P. falciparum* 3D7 strains. Of all the compounds, compound **69** was reported to be the most active antimalarial agents, with an IC_50_ value of 2.6 ± 0.4 nM. Parthiban et al. [151] synthesized a series of chloroquinoline-4H-chromene conjugates with piperazine and azpane rings as tethers, and the determination of their antimalarial activities against 3D7 and K1 strains of *P. falciparum* showed that compounds **70a** and **70b** displayed the most potent antimalarial activity with IC_50_ values between 0.29 and 1.78 µM. Bhat et al. [152] developed a series of hybrid 4-aminoquinoline 1,3,5-triazine derivatives, and an assessment their antimalarial activities showed that compounds **71a** and **71b** were the most active inhibiting chloroquine-sensitive (3D7) and chloroquine-resistant (RKL-2) *P. falciparum* strains with IC_50_ values between 1 and 25 µM. The results of the docking studies, revealed that the most active compounds bound well with *P. falciparum* dihydrofolate reductase thymidylate synthase (*pf*-DHFR-TS). Different oxirane compounds were synthesized by Carneiro et al. [153] and subjected to antimalarial screening against chloroquine-sensitive 3D7 strains of *P. falciparum* after evaluation, compounds **72a** and **72b** were reported to be the most active species among 18 synthesized compounds. Karad et al. [154] synthesized various analogs of morpholinoquinoline-based conjugates with an incorporated pyrazoline ring, and the evaluation of their antimalarial activities in vitro using the biological assay revealed that compounds **73a** and **73b** were the most active antimalarial agents with IC_50_ values between 0.015 and 0.018 µM. Devender et al. [155] synthesized a new series of triazoles and in vitro antimalarial activity evaluation found that among the different compounds synthesized, compounds **74a** and **74b** displayed excellent biological activity against 3D7 and K1 *P. falciparum* strains, with IC_50_ value between 0.3 and 2.11 µM. Svogie et al. [156] prepared a series of indolyl-3-ethanone α-thioethers, and an evaluation of their antimalarial activities revealed that compounds **75a** (IC_50_ = 0.24 µM) and **75b** (IC_50_ = 0.09 µM) were the most active species against 3D7 *P. falciparum* strains. The development of new antimalarial agents, led to the synthesis of a new series of imidazo[4,5-c]quinolin-2-one derivatives by Patel et al. [157] using a four-step synthetic route. The evaluation of an antimalarial activities of derivatives using an allamar-Blue gametocytocidal assay showed that compound **76a** was the most potent candidate among the series. Seebacher et al. [158] prepared a series of several azabicyclic compounds, and an evaluation of their antimalarial activities showed that compounds **77a** and **77b**, with IC_50_ values of 0.28 and 0.095 µM, respectively, showed a remarkable antimalarial activity against 3D7 *P. falciparum* strains. Further analysis proved that compound **77b** was the most potent *P. falciparum* inhibitor of the active species. A new imidazole-based series of substituted ester and carbamate derivatives were synthesized by Vita et al. [115], and an in vitro investigation revealed that compound **78** showed the highest antimalarial effect against K1 *P. falciparum* strains (IC_50_ = 0.6 µM). Inam et al. [159] designed and synthesized several acylhydrazine derivatives, attached to chloroqunoline nuclei with piperazine rings. An evaluation of their antiprotozoal activities revealed that among the compounds, compounds **79a** (IC_50_ = 0.33) and **79b** (IC_50_ = 0.2 µM) were moderately active against w2 strains of *P. falciparum*. Patrick et al. [116] developed a large series of cationic benzyl phenyl ether derivatives for application in antiprotozoal drug development. Their results showed that compounds **80a–c** were extremely potent *P. falciparum* inhibitors, with IC_50_ values of 0.006, 0.004, and 0.006 µM, respectively. The screening and evaluation of the antiprotozoal activities of fifteen 8,4-oxyneolignans analogs prepared by Rye et al. [117], using asymmetric synthesis revealed that compounds **81a–c** showed IC_50_ values between 0.608 and 2.50 µM against *P. falciparum*. Dürüst et al. [118] developed several derivatives of triazoles coupled with 1,2,4-oxadiazole moieties, and an investigation of their antiprotozoal activities revealed that compounds **82a** and **82b** were the most active *P. falciparum* inhibitors, with an IC_50_ values of 13.2 and 14.7 µg/mL, respectively. A new series of metal complexes designed and prepared by Juneja et al. [160] were evaluated for their antimalarial activity against w2 *P. falciparum* strains. The in vitro study revealed that compounds **83a–c** were the best candidates with IC_50_ values between 1.1 and 1.9 µM. McKeever et al. [161] developed aminoalkyl derivatives form guanidine diaromatic minor groove binder, and evaluated their antiprotozoal activities. The results showed that compounds **84a** (IC_50_ = 0.106 µM) and **84b** (IC_50_ = 0.149 µM) elicited a significant *P. falciparum* culture inhibitory. Patrick et al. [120] synthesized thirty-six 4,4″-Diamidino-m-terphenyl analogs, which were tested for their antiprotozoal drug development. Among these 36 compounds, compounds **85a** and **85b**, which bear a dimethyltetrahydropyrimidinyl ring at the para-position, exhibited significant antimalarial activity with IC_50_ values of 0.002 and 0.003 µM, respectively, which were lower than that of their parent drug, chloroquine. From the study, it was evident that a substituent attached at position 4 of a phenyl ring would lead to the exhibition of excellent inhibitory action. Opsenica et al. [162] synthesized a novel series of aminochloroquinoline derivatives, and assessed their antimalarial activities in vivo using different *P. falciparum* strains. The results revealed that compounds **86a–c** were notable antimalarial agents with IC_50_ values between 3.95 and 45.78 nM. Hanessian et al. [163] designed a novel series of pactamycin analogs, and an evaluation of their biological activities found that compounds **87a** (IC_50_ = 3.5 nM) and **87b** (IC_50_ = 6.7 nM) were the most potent molecules against D6 and Dd2 strains of *P. falciparum*. Abada et al. [164] evaluated 24 porphyrin precursors and derivatives against different protozoal strains. Their results showed that compound **88** was the most active member of the series. Its IC_50_ value 0.02 µM was 100 to 200 times lower than that of standard drug chloroquine (15–25 µM). Yeo S J et al. [165] synthesized chloroquine derivatives with phenylmethyl groups and unsaturated amides, which have anti-malarial activity. Among them, compounds **89a** and **89b** showed greater antimalarial activity against 3D7 *P. falciparum* strains with IC_50_ values of 0.17 and 0.23 µM, respectively. Singh et al. [166] synthesized 4-aminoquinolin-ferrocenyl-chalcone derivatives, and the evaluation of their pharmacological properties revealed that compounds **90a–c** were the most potent compounds against W2 strain of *P. falciparum*; their IC_50_ values ranged from 0.37 to 0.53 µM.

In summary, various chemical scaffolds and its analogues such as triazole-tethered chalcone-coumarin hybrids, chloroquinoline-isatin hybrids, flavonones, indolesulfonamides, artemisinin derivatives, *N,N*′-diaryl substituted piperizines, porphyrin derivatives, and polyaryls present significant antimalarial activities. In particular, artemisinin derivatives (**68**) in which lactone was transformed to lactam, then various hydrophilic substituents were introduced, showed good inhibitor activity. As novel scaffolds, symmetric terphenyl cyclic amidines (**85**) exhibited slightly better antimalarial activity than chloroquine and artemisinin.

### 2.4. Anti-Trichomoniasis

Trichomoniasis is a protozoan infection caused by the flagellate protozoan *T. vaginalis.* It is one of the most prevalent nonviral sexually transmitted diseases worldwide [167]. The protozoan *T. vaginalis* affects both men and women. In women, the symptoms of this infection worsen during menstruation whereas in men the infection is largely asymptomatic; these asymptomatic men are considered carriers [168]. Trichomoniasis in men and women is associated with birth outcomes [169], infertility [170], cervical and prostate cancers [171], and pelvic inflammatory disease [172]. In men, this disease is characterized by irritation inside the penis, mild discharge, or slight burning after urination or ejaculation and in women, it is associated with yellow-green vaginal discharge with a strong odor. The infection may also cause discomfort during sex and urination, as well as irritation and itching of the female genital area. In rare cases, lower abdominal pain can occur. The symptoms usually appear in women within 5–28 days of exposure. Generally, the prevalence of *T. vaginalis* infection is found to be higher among women than men. A recent report on trichomoniasis revealed that the prevalence of trichomoniasis in non-human immunodeficiency virus (HIV)-infected persons was 10.1% among women vs. 2.0% among men [173], when compared to HIV-infected women (10%–20% prevalence of trichomoniasis) [174]. Data regarding the prevalence of *T. vaginalis* in men who have sex with men (MSM) are scarce. *T. vaginalis* infection damages the vaginal epithelium, which increases the risk of women being infected by HIV, and thereby considerably increasing the chances of infected women transmitting HIV to her sexual partner(s) [175,176]. In short, *T. vaginalis* is a co-factor in HIV transmission and acquisition [177,178]. According to a WHO report, approximately 248 million new cases of trichomoniasis are reported worldwide annually. It is believed that two to three million symptomatic infections occur annually among sexually active women in the United States [179]. In the Republic of Korea, 10.4% of women complaining of vaginal symptoms and signs were found to be infected with *T. vaginalis* [180].

According to the literature the pathogenicity of *T. vaginalis* is due to cysteine peptidases (CP) enzymes [181,182] which may present on their cell surfaces as secretion products of the parasite [183,184,185,186,187]. These enzymes play a critical role in pathogenicity, as well as in the biological processes of this protozoan. Although, MTZ, which is approved by the FDA is the drug of choice for trichomoniasis, [188] recent studies have shown that this drug has several toxic effects [14,15,16,17,18], and the clinical resistance of many microbes has reduced its efficiency [189,190]. In view of these major drawbacks, there is an urgent need for the development of new and efficient scaffolds against trichomoniasis. Therefore, researchers have designed and synthesized some novel agents as inhibitors of *T. vaginalis* growth. In this review article, we have compiled the latest data (from 2012–2017) on the development of novel antitrichomonial agents. The results are summarized in Table 4.

#### Important Highlights of Table 4 Compounds

Compounds of different class heterocycles are included in Table 4 as potent agents against trichomoniasis and the results of their assessments were compared with those of the standard drugs nitazoxanide, tizoxanide, MTZ, albendazole, and nonoxynol.

In brief, Navarrete-Vázquez et al. [63] synthesized a novel series eight of nitrothiazole and benzothiazole derivatives and among them, compounds **91a** and **91b** showed effective *T. vaginalis* cell growth inhibition, with IC_50_ values of 0.331 and 0.221 µM, respectively. During structure-activity relationship studies of adenosine and uridine analogs against *T. vaginalis*, Shokar et al. [191] reported that compound **92** (IC_50_ = 0.09 µM) was the most potent candidate for trichomoniasis treatment. Interestingly, it has also been approved by the US FDA as a potential drug candidate for trichomoniasis treatment. In a one-step reaction of *N*-substituted β-lactams with a free azide group and 5- substituted isatins containing a terminal alkyne, Raj et al. synthesized a series of β-lactam-isatin-triazole conjugates, and one of them, compounds **93,** was found to be capable of selectively inhibiting *T. vaginalis* growth with an IC_50_ value of 7.06 µM [192]. A novel series of metronidazole-chalcone conjugates were designed and developed by Anthwal et al. [193], and their antitrichomonal activities against a *T. vaginalis* culture were evaluated for in vitro. The results revealed that two of the compounds in the series, compounds **94a** and **94b** were the most effective candidates against both MTZ-susceptible and MTZ-resistant parasite strains. Soria-Arteche et al. [66] synthesized a series of hybrid compounds bearing nitazoxanide and *N*-methylbenzimidazole moieties using different reagents, and the evaluation of their antiprotozoal activities revealed that among the 13 synthesized molecules, compound **95** (IC_50_ = 0.023 µM) displayed greater antitrichomonal activity against *T. vaginalis* compared to those of the other 12 compounds. Several hybrid analogs bearing tetrazole and chromane as bioactive scaffolds were synthesized by Cano et al. [62] using the one pot Ugi-azide multicomponent reaction in the presence of InCl_3_ as catalyst. Thereafter, they were screened, and their antiprotozoal activities were evaluated and among them, compound **96** was identified as the most potent antitrichomonal agent against *T. vaginalis* with an IC_50_ value of 83.9 µM. Nisha et al. [194] prepared a series of *N*-propargylated-isatin Mannich derivatives as potential antitrichomonal agents, using the one-pot CuCl-catalyzed Mannich-type reaction. An evaluation of their activities against *T. foetus* revealed that three of the synthesized compounds **97a–c** elicited promising inhibitory activity on *T. foteus* culture growth, with IC_50_ values between 11.3 and 24.5 µM. In order to develop novel and effective antitrichomonal agents, a series of mono- and bis-uracil-isatin conjugates were prepared using single-step synthetic procedure. After they were evaluated against a *T. vaginalis* culture. Kumar et.al [195] found that compounds **98a** (IC_50_ = 9.86 µM) and **98b** (IC_50_ = 9.79 µM) were the best candidate. Using a single-step CuCl-catalyzed Mannich-type reaction, Nisha et al. [196] synthesized a large series of Mannish-based compounds in which isatin and 4-aminoquinoline ring are linked to the piperazine nucleus. An evaluation of their antitrichomonal activities revealed that compound **99** was the most potent agent against *T. vaginalis* with an IC_50_ value of 23 µM. A series of hybrid conjugates with incorporated β-lactone, triazole, and isatin nuclei were designed and prepared by Raj et al. [197] as as novel *T. vaginalis* inhibitors. The evaluation of the antitrichomonal activities of these compounds in-vitro demonstrated that compounds **100a** and **100b** were the most active agents inhibiting the growth of a *T. vaginalis* culture. Saleh et al. [198] synthesized a new series of hybrid compounds bearing 5-nitrothiazole moiety, and an in vitro assessment of their antiprotozoal activities, and consequently compound **101** displayed the most promising biological activity against *T. vaginalis* strains, with an IC_50_ value of 4.3 µg/mL. In a study aimed at developing of antiparasitic agents, Adams et al. [199] prepared thiosemicarbazone-derived ruthenium metal complexes, and after evaluating their inhibitory properties against the in vitro growth of a G3 *T. vaginalis* strain, they found that the compounds **102a** (IC_50_ = 5.47 µM) and **102b** (IC_50_ = 7.56 µM) displayed the most potent antitrichomonal activities. A novel combinatorial library of β-amino alcohol-based β-lactam–isatin chimeras were designed and developed by Nisha et al. [201], and an evaluation of their potential against *T. vaginalis* in vitro demonstrated that among the synthesized compounds, compound **103** possessed significant antitrichomonal activity, with an IC_50_ value of 9.73 µM. Stringer et al. [201] prepared a series of rhodium metal complexes and analyzed them for their antiprotozoal activity. The results showed that compound **104** displayed an IC_50_ value of 4.80 µM against a *T. vaginalis* culture. A novel series of coumarin-glyoxal hybrid compounds were synthesized by Gupta et al. [202], and their inhibitory activities against *T. vaginalis*, as well as their spermicidal activities were evaluated. The results led to the conclusion that compounds **105a–c** displayed the most potent trichomonacidal activity.

### 2.5. Anti-Trypanosomiasis

Parasitic infections caused by trypanosomatids constitute a major health problem in countries where poor sanitary conditions are prevalent. Diseases acquired by such infections are considered ‘neglected’ because they receive limited funding for the research and development of new treatments. Amongst the neglected tropical diseases (NTD), human African trypanosomiasis (HAT) is endemic throughout sub-Saharan Africa, while American trypanosomiasis (Chagas disease) affects populations in South and Central America.

#### 2.5.1. HAT/African Sleeping Sickness

**Figure 4:**
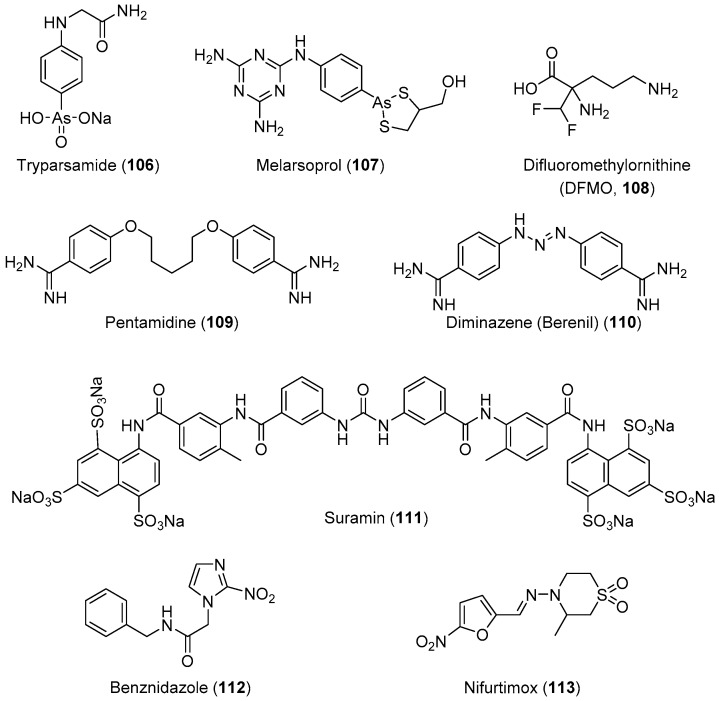
Currently available antitrypanosomal drugs (use in medical practice).

HAT, also known as African sleeping sickness, is one of the most neglected diseases in regions of sub-Saharan Africa; it affects 70 million people in 36 countries. HAT is a vector-borne disease caused by the protozoa parasite *Trypanosoma brucei* [203,204,205]. It is transmitted through the bite of an infected tsetse fly or passed from an infected mother to her child through the placenta. This disease has two stages: the first stage involves parasite-included seizures of the hemolymphatic system, and the second involves the transmission of the parasites into the central nervous system (CNS) across the blood–brain barrier [206]. Infection of the CNS leads to a number of symptoms including mental impairment, severe headaches, fever, chronic encephalopathy, and eventual death. The development of effective vaccines would be an option for preventing this deadly disease; however, trypanosomes can evade the host immune’s system because of the high degree of antigenic variation in glycoproteins forming their surface coat [207,208,209,210,211,212]. Therefore, chemotherapy remains the only viable strategy for the treatment and control of infection. The current chemotherapy for HAT comprises only four drugs. Three of these drugs, suramin, pentamidine, and melarsoprol (Figure 4), were developed over 60 years ago, and exhibit severe side effects. Melarsoprol is an arsenical derivative used for the treatment of HAT in the neurological stage, and they have many undesirable or fatal (3%–10%) side effects in addition to the development of drug resistance, exhibiting a drug failure rate of up to 30%. Pentamidine and suramin are used for treatment in the early stage of the disease, before the involvement of the CNS. Nevertheless, they have many severe side effects such as low blood pressure, decreased level of consciousness, kidney problems, low blood cell levels, and wheezing [30]. Moreover, eflornithine, which is less toxic, is only effective against *T. brucei gambiense* subspecies [213]. Treatment with a combination of nifurtimox and eflornithine is less toxic, but ineffective against the *T. brucei rhodesiense* subspecies.

#### 2.5.2. Chagas Disease

American trypanosomiasis (Chagas disease) is caused by the flagellate parasitic protozoan *T. cruzi*. This infection was first discovered by the Brazilian physician Carlos Chagas (1879−1934) in 1909. While *Trypanosoma cruzi* is transmitted to animals and humans via insect vectors of the Triatominae, a subfamily of the Reduviidae family of insects, commonly known as “kissing bugs” [214], outbreaks of chagas disease are usually caused by foodborne *T. cruzi* since it produces a very aggressive acute form which is often missed by clinicians. This infection is one of the most threatening diseases in Central and South America. A large number of people, approximately 18 million annually, are infected with this parasite. Among them, 50,000 died owing to heart failure. This disease has become a major public health problem in 22 developing countries in Latin America, where more than eight million people suffer from this infection annually [215]. As depicted in its life cycle, the infection is transmitted by two predominant modes: The first is vectorial, through the infected feces/urine of triatomine bugs, and the second is by blood transfusion: the metacyclic trypomastigotes are released in the feces of the insect vector as it takes a blood meal, and they enter the bloodstream via a bite wound or mucosal membrane. Once inside the host, the metacyclic trypomastigotes invade nearby cells and differentiate into their intracellular amastigote form, which multiplies by binary fission. The transformation into trypomastigotes occurs prior to the release from the cells back into the bloodstream, proliferating the infection cycle. Other forms of transmission include congenital, blood transfusion, and contaminated food, to a lesser extent. This disease is endemic to Latin America, affecting people from Mexico to Argentina [216]. The origin and development of this disease is divided into three phases: The (i) acute (short), (ii) latent (long lasting), and (iii) chronic (very serious condition) phases. In the chronic phase, symptoms such as cardiomyopathy and malformation of the intestines (e.g., megaesophagus and megacolon) have been reported. The administration of the antiparasitic drugs benznidazole and nifurtimox is 100% effective in the short acute phase, and both drugs act through the generation of free radicals that kill the parasite. If no treatment occurs at this stage, then this infection silently progresses into the chronic phase in which internal organs such as the heart, peripheral nervous system, esophagus, and colon are irreversibly affected. Benznidazole and nifurtimox are no longer efficient in the chronic phase, and the health of the patients deteriorates rapidly leading to death, usually due to heart failure. Therefore, the development of new antichagasic drugs is of the utmost importance and urgency [216]. In Table 5, we compiled the latest data (2012–2017) on drug development against trypanosomiasis.

##### Important Highlights of Table 5 Compounds

Compounds of different class heterocycles are included in Table 5 as potent agents against *Trypanosoma species* and the results of their assessments were compared to those of standard drugs Tryparsamide, melarsoprol, difluoromethylornitithine, pentamidine, diminazene, suramin, benznidazole and nifurtimox. Among the substituted benzothiophene derivatives developed by Bhambra et al. [217], compound **14a** and **114b** were found to be the most active candidates for HAT treatment with IC_50_ values of 0.60 and 0.53 µM, respectively. In another series of imidazole compounds synthesized by Trunz et al. [218], compounds **115a** and **115b** demonstrated good potency against *T.b.rhodesiense* and their reported IC_50_ values were 0.16 and 0.10 µM, respectively. Bouchikhi et al. [219] designed a series of compounds based on glycosyl-isoindigo conjugates, and an evaluation of their antitrypanosomal activities revealed that among the compounds synthesized, compounds **116a–c** expressed significant biological activity against *T. b. brucei* strains with IC_50_ values between 0.51 and 0.84 µM. Among the several halonitrobenzamides derivatives synthesized by Hwang et al. [220], and evaluated against a *T. b. brucei* culture compounds, compound **117** was found to be the most potent inhibitor (IC_50_ = 1.5 µM). An assessment of the antiprotozoal activities (antitrypanosomal) of a series of nitroimidazole analogs developed by Samant et al. [221] revealed that among the synthesized molecules, compound **118** was the most active antitrypanosomal agent (IC_50_ = 0.25 µM). Ferrins et al. [222] synthesized various anilides and the evaluation of their antitrypanosomal activity revealed that among the compounds synthesized, compound **119** (IC_50_ = 0.091 µM) significantly inhibited a *T. b. rhodesiense* culture. A large series of convolutamine analogs were prepared by Pham et al. [223]. Basically, convolutamine, which is a natural product, is a highly effective antitrypanosomal. All the synthesized convolutamine derivatives were screened against *T. b. brucei* culture, and compounds **120a** and **120b** were reported as the most potent inhibitors with IC_50_ values of 0.7 µM and 0.5 µM, respectively. Samant et al. [224] synthesized a big library of naphthoquinone derivatives, and the evaluation of their antiprypanosomal activities in vitro showed that compounds **121a** (IC_50_ = 0.07 µM) and **121b** (IC_50_ = 0.05 µM) were the most potent compounds in the series. Papadopoulou et al. [225] synthesized a series of azole-based compounds. An assessment of their antitrypanosomal potentials revealed that compounds **122a** and **122b** were the most active agents with IC_50_ values against *T. b. rhodesiense* and *T. cruzi* vetween 0.187 and 0.373 µM. Papadopoulou et al. [226] also designed and developed nitrotriazole-based amide derivatives; an investigation of their antitrypanosomal activities against *T. cruzi* revealed that among them compound **123** was the most potent agent against *T. cruzi* (IC_50_ = 0.008 µM). A series of heterocyclic spiro compounds synthesized by Zelisko et al. [227] were evaluated for their antitrypanosomal activities. The results showed that compound **124** was the most active molecule (IC_50_ = 0.2624 µM) against *T. b. rhodesiense*. An in vitro assessment of the inhibitory effect of a novel series of β-carboline analogs prepared by Manda et al. [98] against *T. b. brucei* revealed that compound **125** was the most potent inhibitory agent among the compounds (IC_50_ = 1.01 µM). Alves et al. [228] developed several semicarbazone derivatives, and evaluated their antiprotozoal activities in vitro by testing their potential to inhibit biological strains. Compound **126** (IC_50_ = 8.5 µM) appeared to be the most potent *T. cruzi* culture inhibitor, with an IC_50_ values of 8.5 µM. Several azabicyclo nonane type derivatives synthesized by Seebacher et al. [158] were screened for their anti-protozoal activity. The results showed that among them, compounds **127a** (IC_50_ = 0.061 µM) and **127b** (IC_50_ = 0.065 µM) exhibited the greatest activity profiles. Upadhayaya et al. [229] developed a series of quinolone- and indenoquinoline-based heterocycles, and an evaluation of their antiprotozoal potentials revealed that compound **128** was a potential candidate against *T. cruzi* and *T. b. rhodesiense* with IC_50_ values of 0.25 and 1.81 µM, respectively. Vita et al. [115] synthesized series of potent and effective imidazole incorporated phenylethanol derivatives, of which compound **129** exhibited the highest antitrypanosomal activity (IC_50_ = 0.04 µM), and showed more potency against *T. cruzi* strains. Martínez et al. [230] reported the synthesis of several bisguanidine *T. b. rhodesiense* strain inhibitors among which compound **130** (IC_50_ = 0.009 µM) was identified as the most active agent for trypanosomiasis treatment. Patrick et al. [116] developed a new series of benzyl phenyl ether diamidine derivatives, and investigated their potential against a *T. b. rhodesiense* culture. Compound **131**, which exhibited a good therapeutic potential (IC_50_ = 0.003 µM), was identified as the best candidate. Dürüst et al. [118] synthesized a new series of 1,2,4-oxadiazole-linked triazole derivatives using the 1,3-dipolar cycloaddition reaction, and their antiprotozoal activities were analyzed. Of these compounds, compound **132** was identified as the most potent antitrypanosomal agents (IC_50_ = 7.0 µM). As already reported, Mckeever et al. [161] developed a series ofguanidine diaromatic minor grove binder aminoalkyl derivatives. They did not only evaluate the antimalarial activities of these compounds, they also evaluated their antitrypnosomal properties and found that compounds **133a** (IC_50_ = 13.1 µM) and **133b** (IC_50_ = 20.2 µM) were the most potent candidates against *T. b. rhodesiense* strains. A series of 1,3-dipyridylbenzene derivatives were evaluated for their antitrypanosomal activity. The results showed that compounds **134a** and **134b** were the best candidates against *T. b. rhodesiense* and *T. cruzi* strains [120]. Sola et al. [231] synthesized a novel series of huprine Y dimer derivatives, and assessment of their antiprotozoal activities using a bioassay showed that compounds **135a** (IC_50_ = 0.52 µM) and **135b** (IC_50_ = 0.57 µM) were the most significant *T. brucei* culture inhibitors.

## 3. Conclusions

Infections caused by protozoan parasites such as giardiasis, leishmaniasis, malaria, trichomoniasis, and trypanosomiasis are responsible for considerable morbidity and mortality worldwide, with devastating social and economic consequences. The currently available drugs for the treatment of and protection against protozoan parasites were discovered over 50 years ago, and a number of factors limit their utility, such as high cost, poor compliance, drug resistance, low efficacy, and safety concerns. Therefore, the development of new and more effective drugs with fewer side effects presents a crucial challenge. Currently, research focused on the developing new drugs to protect against and treat protozoans are increasing steadily. In this review article, we have presented some of the developments in this field, with the aim of showing the recent significant advances in the discovery of new antiprotozoal drugs.

## Figures and Tables

**Table 1:** Selected data of reported antigiardial agents.

Compound	Activity		Ref.
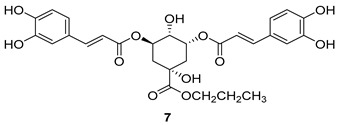		*G. lamblia* [IC_50_ (µg/mL)]	[61]
	**7**	4.62 ± 0.12	
	MTZ	0.86 ± 0.03	
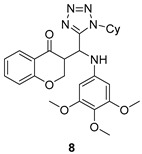		*G. lamblia* [IC_50_ (µg/mL)]	[62]
	**8**	84.2	
	MTZ	1.22	
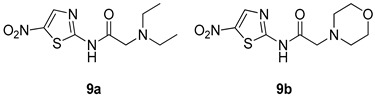		*G. intestinalis* [IC_50_ (µM)]	[63]
	**9a**	0.122	
	**9b**	0.151	
	Aminitrozole	0.490	
	MTZ	5.360	
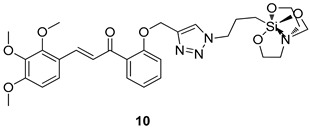		*G. lamblia* [IC_50_ (µM)]	[64]
	**10**	19.58	
	MTZ	62.48	
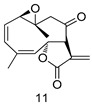		*G. lamblia* [IC_50_ (µg/mL)]	[65]
	**11**	30.6	
	MTZ	0.21	
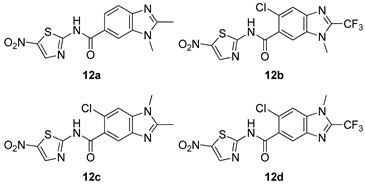		*G. intestinalis* [IC_50_ (µM)]	[66]
	**12a**	0.027 ± 0.002	
	**12b**	0.022 ± 0.001	
	**12c**	0.027 ± 0.002	
	**12d**	0.021 ± 0.005	
	Nitazoxamide	0.015 ± 0.002	
	Mebendazole	0.046 ± 0.009	
	MTZ	1.224 ± 0.021	
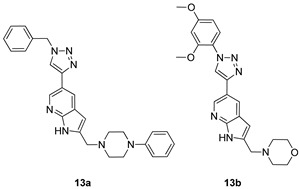		*G. duodenalis* [IC_50_ (µg/mL)]	[67]
	**13a**	14.3	
	**13b**	8.2	
	MTZ	11.4	
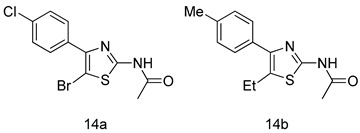		*G. intestinalis* [IC_50_ (µM)]	[68]
	**14a**	0.87	
	**14b**	0.39	
	MTZ	1.40	
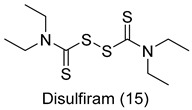		*G. lamblia* [IC_50_ (µM)]	[69]
	Disulfiram (**15**)	6.6	

**Table 2:** Selected data of reported antileishmanial agents.

Compound	Activity			Ref.
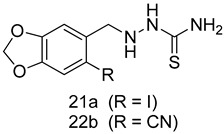		*L. amazonensis* (Promastigotes) [IC_50_ (µM)]	*L. amazonensis* (Amastigotes) [IC_50_ (µM)]	[94]
	**21a**	20.74 ± 0.5	22.0 ± 0.1	
	**22b**	16.4 ± 1.1	17.0 ± 1.1	
	Pentamidine	4.8 ± 0.1	1.9 ± 0.1	
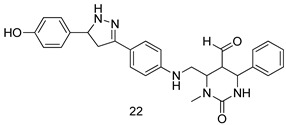		*L. major* [IC_50_ (µg/mL)]	*L. donovani* [IC_50_ (µg/mL)]	[95]
	**22**	0.47 ± 0.02	1.5 ± 0.17	
	AmB	0.56 ± 0.01	-	
	SSG	-	2.98	
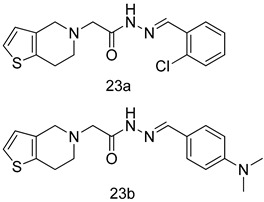		*L. donovani* [IC_50_ (µg/mL)]		[96]
	**23a**	98.75		
	**23b**	93.75		
	SSG	490		
	Pentamidine	5.5		
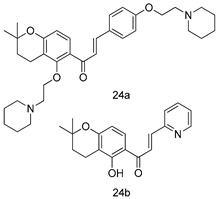		*L. donovani* (Amastigotes) [IC_50_ (µM)]		[97]
	**24a**	2.8		
	**24b**	2.0		
	SSG	49.7		
	Miltefosine	8.4		
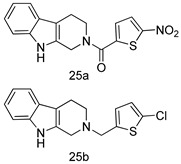		*L. donovani* [IC_50_ (µM)]		[98]
		Promastigotes		
	**25a**	12.7		
	**25b**	9.1		
	Pentamidine	4.8		
	AmB	0.3		
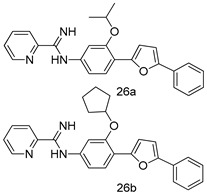		*L. amazonensis* [IC_50_ (µM)]	*L. donovani* [IC_50_ (µM)]	[99]
	**26a**	0.13	0.31	
	**26b**	0.14	0.17	
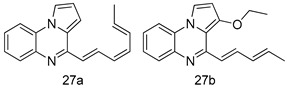		*L. major* [IC_50_ (µM)]	*L. donovani* [IC_50_ (µM)]	[100]
	**27a**	1.2 ± 0.7	10.5 ± 0.6	
	**27b**	1.5 ± 1.1	4.0 ± 0.6	
	Pentamidine	4.3 ± 0.8	5.5 ± 0.8	
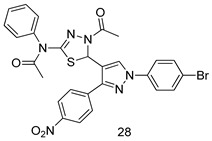		*L. aethiopica* [IC_50_ (µg/mL)]		[101]
		Promastigotes	Amastigotes	
	**28**	0.0142 ± 0.004	0.13 ± 0.02	
	Miltefosine	3.19 ± 14	0.3 ± 0.04	
	AmB	0.0472 ± 0.002	0.2 ± 0.02	
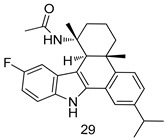		*L. infantum* [IC_50_ (µM)]		[102]
	**29**	1.5 ± 0.1		
	Miltefosine	3.4 ± 0.6		
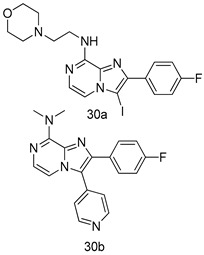		*L. major* [IC_50_ (µM)]		[103]
		Promastigotes	Amastigotes	
	**30a**	2.8 ± 0.4	0.2 ± 0.1	
	**30b**	6.4 ± 0.2	0.8	
	Pentamidine	4.6 ± 1.1	3 ± 1	
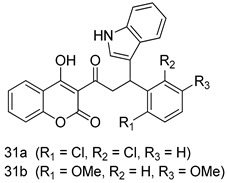		*L. major* [IC_50_ (µg/mL)]		[104]
	**31a**	95.00		
	**31b**	99.00		
	SSG	490.00		
	Pentamidine	5.50		
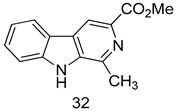		*L. donovani* (Promastigotes) [IC_50_ (µM)]		[105]
	**32**	9.0 ± 2.80		
	Miltefosine	11.9 ± 2.70		
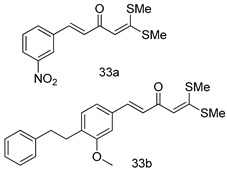		*L. donovani* (Antiamastigotes) [IC_50_ (µM)]		[106]
	**33a**	5.12		
	**33b**	3.56		
	Miltefosine	12.50		
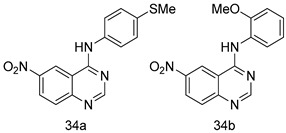		*L. major* [IC_50_ (µM)]		[107]
	**34a**	1.87 ± 0.31		
	**34b**	4.37 ± 0.02		
	Pentamidine	5.09 ± 0.09		
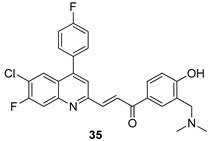		*L. donovani* [IC_50_ (µM)]		[108]
	**35**	0.84 ± 0.12		
	Miltefosine	8.10 ± 0.60		
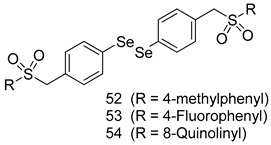		*L. infantum* [IC_50_ (µM)]		[109]
	**36a**	1.40 ± 0.09		
	**36b**	1.47 ± 0.07		
	**36c**	0.83 ± 0.04		
	Miltefosine	2.84 ± 0.10		
	Edelfosine	0.82 ± 0.13		
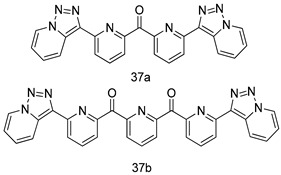		*L. infantum* [IC_50_(µM)]	*L. amazonesis* [IC_50_(µM)]	[110]
	**37a**	19.5	114.6	
	**37b**	38.0	inactive	
	Miltefosine	23.7	20.9	
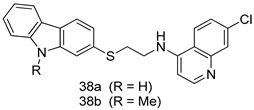		*L. donovani* [IC_50_ (µM)]		[111]
		Promastogote	Amastogote	
	**38a**	8.57 ± 1.58	1.11 ± 0.19	
	**38b**	6.27 ± 0.65	0.36 ± 0.10	
	Miltefosine	1.10 ± 0.26	8.10 ± 1.41	
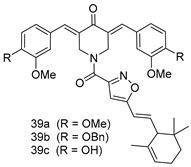		*L. donovani* [IC_50_ (µM)]		[112]
	**39a**	3.75 ± 0.31		
	**39b**	5.02 ± 0.49		
	**39c**	1.83 ± 0.21		
	Miltefosine	8.10 ± 0.51		
	SSG	53.12 ± 4.56		
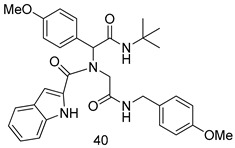		*L. donovani* Amastigote [IC_50_ (µM)]		[113]
	**40**	0.6 ± 0.2		
	Miltefosine	8.4 ± 1.2		
	SSG	56.1 ± 3.2		
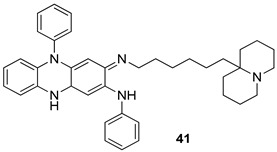		*L. infantum* [IC_50_ (µM)]	*L. tropica* [IC_50_ (µM)]	[114]
	**41**	0.23 ± 0.05	0.12 ± 0.03	
	Clofazimine	4.48 ± 1.06	2.96 ± 1.23	
	AmB	0.08 ± 0.02	0.09 ± 0.04	
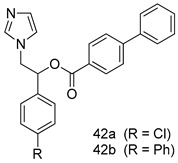		*L. infantum* [IC_50_ (µM)]		[115]
	**42a**	12.7		
	**42b**	8.0		
	Miltefosine	10.4		
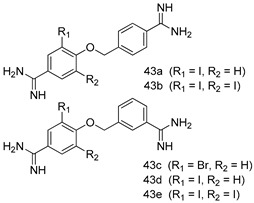		*L. donovani* [IC_50_ (µM)]		[116]
	**43a**	1.94		
	**43b**	1.6		
	**43c**	1.86		
	**43d**	1.27		
	**43e**	1.39		
	Pentamidine	1.84		
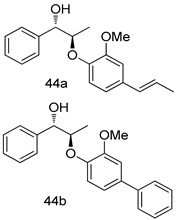		*L. donovani* [IC_50_ (µg/mL)]		[117]
	**44a**	2.29		
	**44b**	2.48		
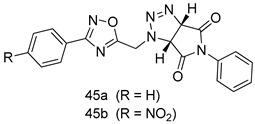		*L. donovani* [IC_50_ (µg/mL)]		[118]
	**45a**	1.6		
	**45b**	2.0		
	Miltefosine	0.171		
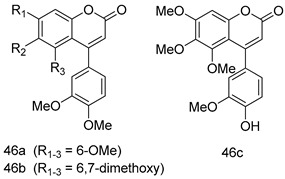		*L. donovani* (Amastigotes) [IC_50_ (µM)]		[119]
	**46a**	5.4		
	**46b**	1.1		
	**46c**	2.4		
	AmB	0.10		
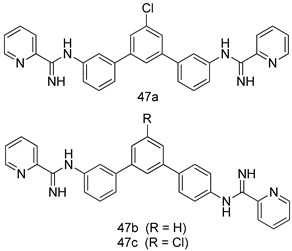		*L. amazonensis* [IC_50_ (µM)]		[120]
	**47a**	0.095		
	**47b**	0.123		
	**46c**	0.211		
	AmB	0.124		
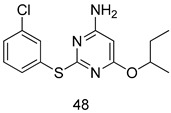		*L. infantum* [IC_50_ (µM)]		[121]
	**48**	29.43 ± 1.34		
	AmB	0.52 ± 1.34		
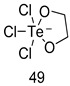		*L. donovani* [IC_50_ (µM)]		[122]
		Promastigotes	Amastigotes	
	**49**	26.9	10.6	
	Miltefosine	6.1	8.2	
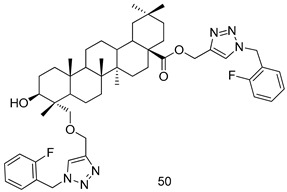		*L. infantum* [IC_50_ (µM)]		[123]
	**50**	5.6 ± 0.14		
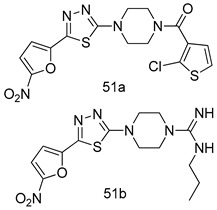		*L. major* [IC_50_ (µM)]		[124]
		Promastigotes	Amastigotes	
	**51a**	9.35	2.7	
	**51b**	0.08	-	

**Table 3:** Selected data of reported antimalarial agents.

Compounds	Activity			Ref.
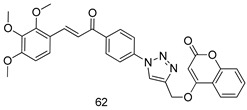		*P. falciparum* [IC_50_ (µM)]		[143]
	**62**	1.60		
	Dihydro-artemisinin	0.0011		
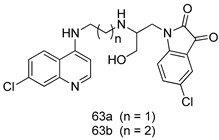		*P. falciparum* [IC_50_ (nM)]		[144]
	**63a**	11.7		
	**63b**	13.5		
	Chloroquine	36.37		
	Artemisinin	4.37		
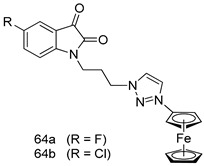		*P. falciparum* [IC_50_ (µM)]		[145]
		3D7 strain	W2 strain	
	**64a**	3.76	5.97	
	**64b**	8.49	4.58	
	Chloroquine	0.021	0.49	
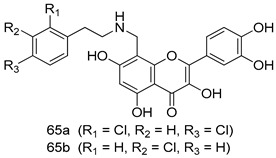		*P. falciparum* [IC_50_ (µM)]		[146]
		W2 strain	D6 strain	
	**65a**	0.071	0.065	
	**65b**	0.079	0.069	
	Artemisinin	0.007	0.009	
	Chloroquine	0.63	0.014	
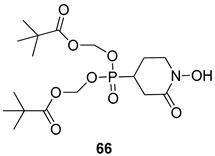		*P. falciparum* [IC_50_ (nM)]		[147]
	**66**	27.4		
	Chloroquine	300		
	Mefloquine	92		
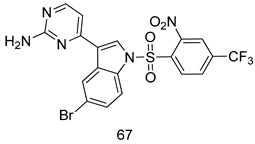		*P. falciparum* [IC_50_ (µM)]		[148]
		D6 strain	W2 strain	
	**67**	2.56	3.41	
	Artemisinin	<0.09	<0.09	
	Chloroquine	<0.08	0.72	
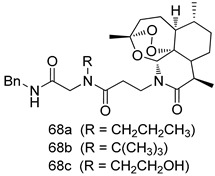		*P. falciparum* [IC_50_ (nM)]		[149]
	**68a**	0.3		
	**68b**	0.7		
	**68c**	1.5		
	Artemisinin	19		
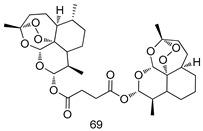		*P. falciparum* [IC_50_ (nM)]		[150]
	**69**	2.6 ± 0.4		
	Chloroquine	9.8 ± 2.8		
	Dihydro-Artemisinin	1.1 ± 0.5		
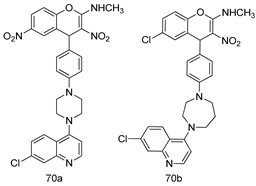		*P. falciparum* [IC_50_ (µM)]		[151]
		3D_7_	K1	
	**70a**	0.62	1.78	
	**70b**	0.29	0.496	
	Chloroquine	0.005	0.254	
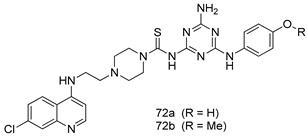		% Dead asexual parasites (*P. falciparum*)		[152]
		3D_7_	RKL-2	
	**71a**	25.7	5.0	
	**71b**	20.0	1.0	
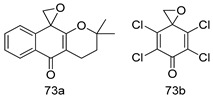		*P. falciparum* [IC_50_ (µM)] 3D_7_ strain		[153]
	**72a**	3.71		
	**72b**	3.95		
	Chloroquine	0.18		
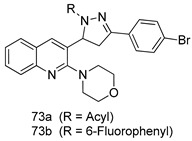		*P. falciparum* [IC_50_ (µM)]		[154]
	**73a**	0.018		
	**73b**	0.015		
	Chloroquine	0.062		
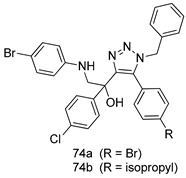		*P. falciparum* [IC_50_ (µM)]		[155]
		3D_7_	K_1_	
	**74a**	0.87	2.04	
	**74b**	0.3	2.11	
	Chloroquine	0.011	1.2	
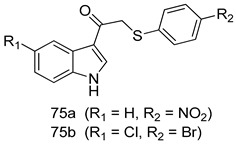		*P. falciparum* [IC_50_ (µM)] 3D7 strain		[156]
	**75a**	0.24		
	**75b**	0.09		
	Chloroquine	0.028		
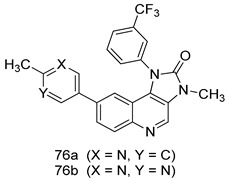		*P. falciparum* [IC_50_ (µM)] 3D_7_ strain		[157]
	**76a**	0.007		
	**76b**	0.017		
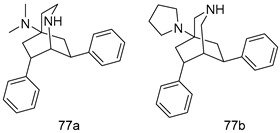		*P. falciparum* [IC_50_ (µM)] K_1_ strain		[158]
	**77a**	0.28		
	**77b**	0.095		
	Chloroquine	0.12		
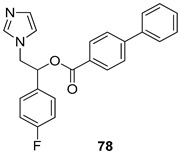		*P. falciparum* [IC_50_ (µM)] K_1_ strain		[115]
	**78**	0.6		
	Chloroquine	0.14		
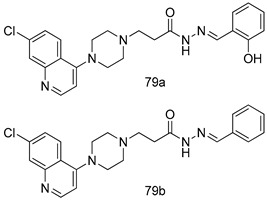		*P. falciparum* [(IC_50_ µM)] W2 strain		[159]
	**79a**	0.33 ± 0.095		
	**79b**	0.2 ± 0.05		
	Mefloquine	0.04 ± 0.01		
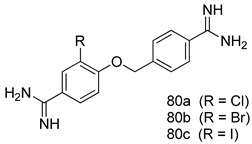		*P. falciparum* [IC_50_ (µM)]		[116]
	**80a**	0.006		
	**80b**	0.004		
	**80c**	0.006		
	Chloroquine	0.201		
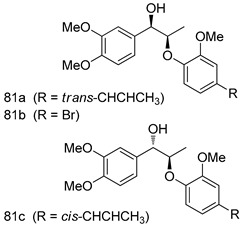		*P. falciparum* [IC_50_ (µg/mL)]		[117]
	**81a**	0.608		
	**81b**	2.50		
	**81c**	1.71		
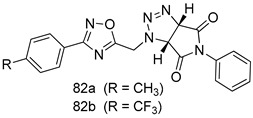		*P. falciparum* [IC_50_ (µg/mL)]		[118]
	**82a**	13.2		
	**82b**	14.7		
	Chloroquine	0.073		
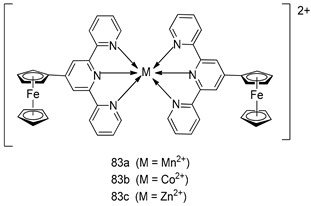		*P. falciparum* [IC_50_ (µM)]		[160]
	**83a**	1.1 ± 1.1		
	**83b**	1.9 ± 0.9		
	**83c**	1.5 ± 0.14		
	Mefloquine	0.004 ± 0.02		
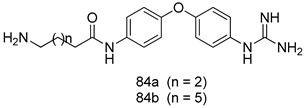		*P. falciparum* [IC_50_ (µM)]		[161]
	**84a**	0.106		
	**84b**	0.149		
	Chloroquine	0.0039		
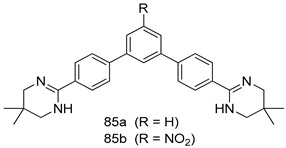		*P. falciparum* [IC_50_ (µM)]		[120]
	**85a**	0.002		
	**85b**	0.003		
	Chloroquine	0.125		
	Artemisinin	0.004		
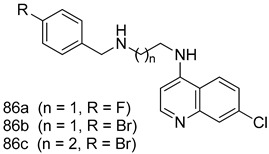		*P. falciparum* [IC_50_ (nM)]		[162]
		W2	D6	
	**86a**	45.78	26.38	
	**86b**	9.47	4.10	
	**86c**	5.93	3.95	
	Artemisinin	6.7	9.00	
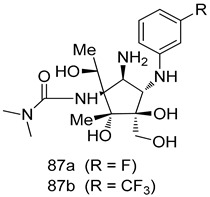		*P. falciparum* [IC_50_ (nM)]		[163]
		D6	Dd2	
	**87a**	6.5	7.4	
	**87b**	6.7	3.5	
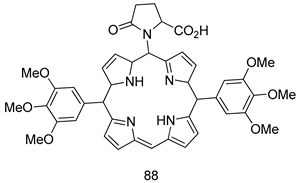		*P. falciparum* [IC_50_ (µM)]		[164]
	**88**	0.02		
	Chloroquine	15~25		
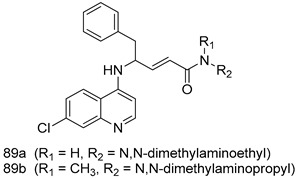		*P. falciparum* [IC_50_ (µM)]		[165]
	**89a**	0.17 ± 0.01		
	**89b**	0.23 ± 0.01		
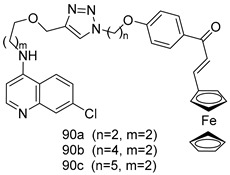		*P. falciparum* [IC_50_ (µM)]		[166]
	**90a**	0.43 ± 0.02		
	**90b**	0.53 ± 0.04		
	**90c**	0.37 ± 0.03		
	Chloroquine	0.06		

**Table 4:** Selected data of reported antitrichomonal agents.

Compound	Activity			Ref.
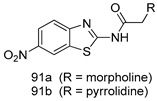		*T. vaginalis* [IC_50_ (µM)]		[63]
	**91a**	0.331		
	**91b**	0.221		
	MTZ	0.290		
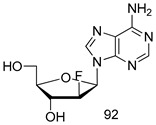		*T. vaginalis* [IC_50_ (µM)]		[191]
	**92**	0.09		
	MTZ	0.72		
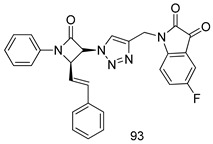		*T. vaginalis* [IC_50_ (µM)]		[192]
	**93**	7.06		
	MTZ	0.72		
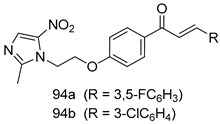		*T. vaginalis* [IC_50_ (µg/mL)]		[193]
		MTZ-susceptible	MTZ-resistant	
	**94a**	1.56	3.125	
	**94b**	1.56	3.125	
	MTZ	1.56	12.5	
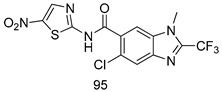		*T. vaginalis* [IC_50_ (µM)]		[66]
	**95**	0.023± 0.005		
	Nitazoxanide	0.107 ± 0.009		
	MTZ	0.213 ± 0.004		
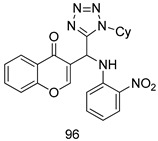		*T. vaginalis* [IC_50_ (µg/mL)]		[62]
	**96**	83.9		
	MTZ	0.037		
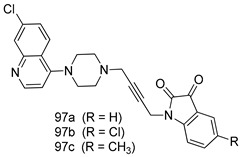		*T.foetus* [IC_50_ (µM)]		[194]
	**97a**	22.2		
	**97b**	11.3		
	**97c**	24.5		
	MTZ	0.72		
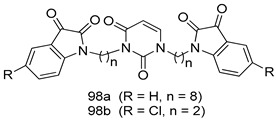		*T. vaginalis* [IC_50_ (µM)]		[195]
	**98a**	9.86		
	**98b**	9.79		
	MTZ	0.72		
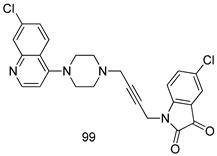		*T. vaginalis* [IC_50_ (µM)]		[196]
	**99**	23		
	MTZ	0.72		
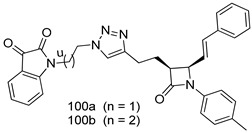		*T. vaginalis* [IC_50_ (µM)]		[197]
	**100a**	10.49 ± 1.05		
	**100b**	25.60 ± 1.08		
	MTZ	0.72		
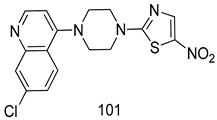		*T. vaginalis* [IC_50_ (µg/mL)]		[198]
	**101**	4.3 ± 1.2		
	MTZ	8.5 ± 0.9		
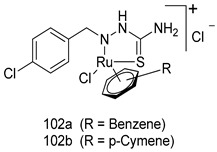		*T. vaginalis* [IC_50_ (µM)]		[199]
	**102a**	5.47		
	**102b**	7.56		
	MTZ	0.72		
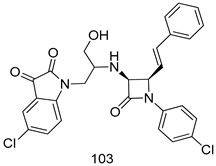		*T. vaginalis* [IC_50_ (µM)]		[200]
	**103**	9.73 ± 1.13		
	MTZ	0.72		
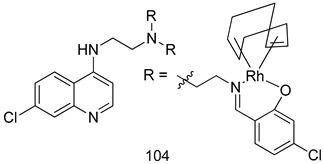		*T. vaginalis* [IC_50_ (µM)]		[201]
	**104**	4.80 ± 0.54		
	MTZ	0.72		
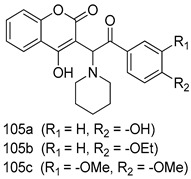		*T. vaginalis* [IC_50_ (µg/mL)]		[202]
		MTZ-susceptible	MTZ-resistant	
	**105a**	0.0294	0.2363	
	**105b**	0.0140	0.1122	
	**105c**	0.0136	0.1091	
	MTZ	0.0182	0.3655	

**Table 5:** Selected data of reported antitrypanosomal agents.

Compound	Activity			Ref.
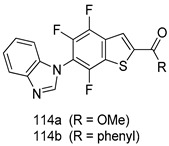		*T. b. rhodesiense* [IC_50_ (µM)]		[217]
	**114a**	0.60		
	**114b**	0.53		
	Melarsoprol	0.051		
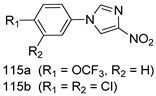		*T. b. rhodesiense* [IC_50_ (µM)]		[218]
	**115a**	0.16		
	**115b**	0.10		
	Melarsoprol	0.009		
	Eflornithine	3.80		
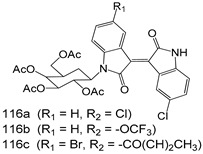		*T. brucei* [IC_50_ (µM)]		[219]
	**116a**	0.84		
	**116b**	0.60		
	**116c**	0.51		
	Melarsoprol	0.013		
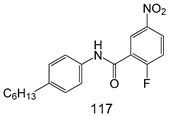		*T. brucei* [IC_50_ (µM)]		[220]
	**117**	1.5 ± 0.4		
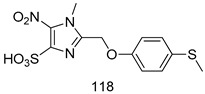		*T. brucei* [IC_50_ (µM)]		[221]
	**118a**	0.25 ± 0.1		
	**118b**	0.67 ± 10.1		
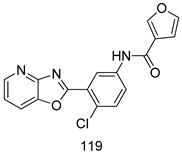		*T. b. rhodesiense* [IC_50_ (µM)]		[222]
	**119**	0.091		
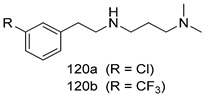		*T. b. brucei* [IC_50_ (µM)]		[223]
	**120a**	0.7		
	**120b**	0.5		
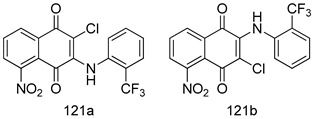		*T. brucei* [IC_50_ (µM)]		[224]
	**121a**	0.07 ± 0.01		
	**121b**	0.05 ± 0.01		
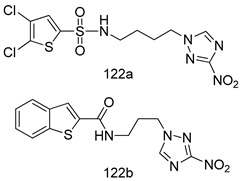		*T. b. rhodesiense* [IC_50_ (µM)]	*T. cruzi* [IC_50_ (µM)]	[225]
	**122a**	0.218	0.373	
	**122b**	0.187	0.239	
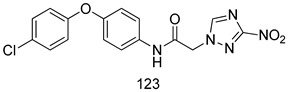		*T. cruzi* [IC_50_ (µM)]		[226]
	**123**	0.008		
	Benznidazole	2.153 ± 0.176		
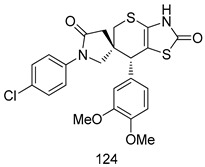		*T. b. brucei* [IC_50_ (µM)]		[227]
	**124**	0.2624 ± 0.0139		
	pentamidine	0.0032 ± 0.0004		
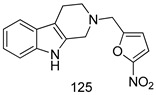		*T. brucei* [IC_50_ (µM)]		[98]
	**125**	1.01		
	Pentamidine	0.0041		
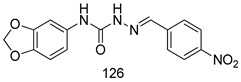		*T. cruzi* [IC_50_ (µM)]		[228]
	**126**	8.5		
	Nifurtimox	7.7		
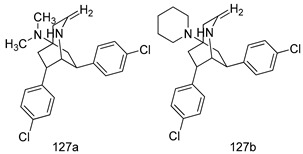		*T. b. rhodesiense* [IC_50_ (µM)]		[158]
	**127a**	0.061		
	**127b**	0.065		
	Melarsoprol	0.0039		
	Suramin	0.0075		
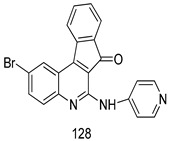		*T. b. rhodesiense* [IC_50_ (µM)]	*T. cruzi* [IC_50_ (µM)]	[229]
	**128a**	1.81	0.25	
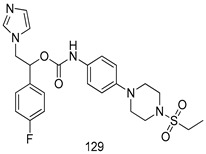		*T. cruzi* [IC_50_ (µM)]		[115]
	**129**	0.04		
	Benznidazole	1.95		
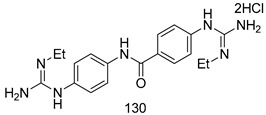		*T. b. rhodesiense* [IC_50_ (µM)]		[230]
	**130**	0.009		
	Melarsoprol	0.008		
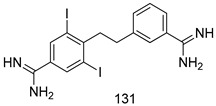		*T. b. rhodesiense* [IC_50_ (µM)]		[116]
	**131**	0.003		
	Pentamidine	0.003		
	Melarsoprol	0.006		
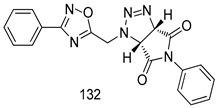		*T. b. rhodesiense* [IC_50_(µg/mL)]		[118]
	**132**	7.0		
	Melarsoprol	0.005		
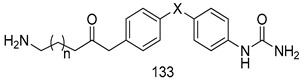		*T. b. rhodesiense* [IC_50_(µM)]		[161]
	**133a**	13.1		
	**133b**	20.2		
	Pentamidine	0.005		
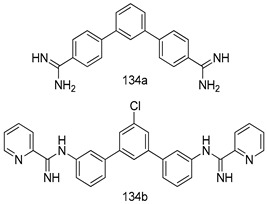		*T. b. rhodesiense* [IC_50_ (µM)]	*T. cruzi* [IC_50_ (µM)]	[120]
	**134a**	0.004	71.6	
	**134b**	0.019	0.053	
	Pentamidine	0.003	-	
	Melarsoprol	0.004	-	
	Benznidazole	-	1.30	
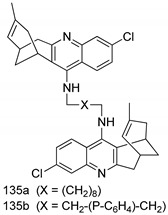		*T. brucei* [IC_50_ (µM)]		[231]
	**135a**	0.52 ± 0.01		
	**135b**	0.57 ± 0.02		

## Abbreviations

HAT	Human African trypanosomiasis
MTZ	Metronidazole
ML	Mucosal leishmaniasis
SSG	Sodium gluconate
AmB	Amphotericin-B
CFM	Clofazimine
EEF	Exoerythrocytic form
RBC	Red blood cell
*pf*-DHFR-TS	*P.falciparum* dihydrofolate reductase thymidylate synthase
CP	Cysteine peptidase
NTD	Neglected tropical disease
CNS	Central nervous system
